# A Unified Model for BDS Wide Area and Local Area Augmentation Positioning Based on Raw Observations

**DOI:** 10.3390/s17030507

**Published:** 2017-03-03

**Authors:** Rui Tu, Rui Zhang, Cuixian Lu, Pengfei Zhang, Jinhai Liu, Xiaochun Lu

**Affiliations:** 1 National Time Service Center, Chinese Academy of Sciences, Shu Yuan Road, Xi’an 710600, China; turui-2004@126.com (R.T.); zhangruiwkk@163.com (R.Z.); zpf.333@163.com (P.Z.); jinhailiu15@163.com (J.L.); lxc@ntsc.ac.cn (X.L.); 2 Key Laboratory of Precision Navigation and Timing Technology, Chinese Academy of Sciences, Shu Yuan Road, Xi’an 710600, China; 3 College of earth sciences, University of Chinese Academy of Sciences, Yu Quan Road, Beijing 100049, China; 4 Germany Research Center for Geosciences, Telegrafenberg, Potsdam 14473, Germany

**Keywords:** BDS, PPP/RTK, local augmentation positioning, ambiguity resolution

## Abstract

In this study, a unified model for BeiDou Navigation Satellite System (BDS) wide area and local area augmentation positioning based on raw observations has been proposed. Applying this model, both the Real-Time Kinematic (RTK) and Precise Point Positioning (PPP) service can be realized by performing different corrections at the user end. This algorithm was assessed and validated with the BDS data collected at four regional stations from Day of Year (DOY) 080 to 083 of 2016. When the users are located within the local reference network, the fast and high precision RTK service can be achieved using the regional observation corrections, revealing a convergence time of about several seconds and a precision of about 2–3 cm. For the users out of the regional reference network, the global broadcast State-Space Represented (SSR) corrections can be utilized to realize the global PPP service which shows a convergence time of about 25 min for achieving an accuracy of 10 cm. With this unified model, it can not only integrate the Network RTK (NRTK) and PPP into a seamless positioning service, but also recover the ionosphere Vertical Total Electronic Content (VTEC) and Differential Code Bias (DCB) values that are useful for the ionosphere monitoring and modeling.

## 1. Introduction

Relative positioning and Precise Point Positioning (PPP) are two basic approaches in precise Global Navigation Satellite System (GNSS) data processing. For the relative positioning, data from a network of stations are analyzed simultaneously to estimate the receiver positions and other parameters such as zenith troposphere delays and the integer-cycle phase ambiguities [1,2]. It shows an advantage in effectively eliminating the common errors derived from the multiple satellites and stations, including the GNSS receiver clock and satellite clock errors. Thus, the integer-cycle phase ambiguities can be easily resolved to their correct integer values to achieve mm-cm level precision in a local reference frame [3]. However, it requires the simultaneous observations for the whole network and the selection of reference stations.

In terms of the PPP technology [4], it relies on fixed satellite orbits and satellite clocks to estimate the positions of receivers, phase ambiguities, zenith troposphere delays, and receiver clock parameters. As no specific reference station is needed, PPP is more flexible and has been widely used in many high-precision areas [5,6,7]. After an initialization of half an hour or longer, the dynamic PPP can obtain a centimeter precision in the global reference. Furthermore, the convergence time and positioning accuracy can be improved by the newly developed PPP ambiguity resolution methods [8,9,10,11]. In addition, the latest development on speeding up PPP initialization can be resolving un-differenced ambiguities for multi-GNSS simultaneously [12,13,14,15].

The Real-Time Kinematic (RTK) positioning is also of great interest for the GNSS community. Generally, the ambiguity fixed solutions can be achieved using a few seconds of measurements. The positioning accuracy is at the centimeter level when the baselines are shorter than a few tens of kilometers [16,17,18]. In the case of longer baselines, a network of Continuously Operating Reference Stations (CORS) can be established to spatially interpolate the atmospheric corrections, and thus the positioning accuracy is significantly improved compared to the RTK [19,20,21]. This service mode is named as Network RTK (NRTK). The deficiencies of NRTK are also obvious. On one hand, the interpolated atmospheric corrections are only effective within the area of the CORS network, as the accuracy of atmospheric corrections is rapidly degraded for users located out of the CORS network [22,23]. On the other hand, due to the high cost of deploying the CORS networks, NRTK can only cover a regional area and hence can hardly evolve into a precise positioning service on the global scale [24]. Accordingly, some new positioning algorithms were developed. Ge et al. proposed the Un-differenced network RTK (URTK), which utilized Double Differenced (DD) observations for the server solutions and Un-differenced (UD) observations for the user solutions [25]. It provided the rovers a rapid and precise absolute positioning service. Teunissen et al. proposed a CORS based PPP-RTK, which made use of UD observations for both the server and rover solutions [26]. URTK is different from the CORS based PPP-RTK, as URTK does not need clock corrections and ionospheric delays for the reference network solution, and URTK’s rover can use precise or broadcast ephemeris products. It is also different from regional reference network-augmented precise point positioning as the DD-ambiguity resolution is more rapid than fixing the integer ambiguity in PPP for the reference network [27], thus shortening the initialization time of modeling the corrections. The main differential corrections between NRTK and URTK are also different. As the NRTK usually uses the Virtual Reference Station (VRS) technique, it will form a virtual observation and broadcast it to the corresponding user, then the user using the standard RTK for the fast positioning [21]. And for the URTK, it usually broadcasts the UD Observed Minus Computed (OMC) corrections from the reference stations. The users then selected and interpolated these corrections and using PPP for the fast positioning [25]. Meanwhile, the URTK is a one-way communication, with the need of receiving the server’s corrections. In addition, it is possible to integrate URTK and PPP into a seamless positioning service [28,29].

Nowadays, some researchers have proposed a new PPP model using the raw observations [11,30,31,32,33], which shows several advantages compared to the ionospheric-free observations [34]. Firstly, it will reduce the observing noise and increase the number of the observations. Secondly, the ionosphere and Differential Code Bias (DCB) information can be recovered. Thirdly, it can be used for the single-frequency users. For example, Gu et al. discussed the BeiDou Navigation System (BDS) PPP with raw observations and tried to resolve the un-differenced ambiguities [35]. However, as most of the previous studies mainly focused on (Global Positioning System) GPS, GLObalnaya NAvigatsionnaya Sputnikovaya Sistema (GLONASS) and BDS PPP, further investigations concerning the BDS NRTK using raw observations are still needed.

So far, 14 BDS satellites (five Geostationary Earth Orbit (GEO), five Inclined Geosynchronous Orbit (IGSO), four Mid Earth Orbit (MEO)) are in orbit and providing positioning service for the Asian-Pacific region [33,36,37,38]. Besides, the BDS ground-based augmentation system is also under construction. It consists of 150 base stations for providing the NRTK and/or the PPP service in China. Therefore, the study of the unified model for BDS wide area and local area augmentation positioning based on raw observations is of crucial significance, not only for the integration of PPP and NRTK but also for geosciences application.

In this contribution, we proposed a unified model for BDS wide area and local area augmentation positioning based on raw observations. First of all, the separated PPP and/or URTK model with raw observations are introduced. Secondly, the integrated model of PPP and/or URTK with the raw observations is presented. Then, the experimental BDS data are applied for the purpose of evaluation and validation. Finally, the conclusions and discussion are provided.

## 2. The PPP Solution Model Based on Raw Observations

Generally, the ionospheric-free combinations with dual-frequency observations are applied to eliminate the first-order ionospheric delays, which, however, will lead to the increase of the measurement noise. In this study, as the raw observations are used, the ionospheric delays are estimated as unknown parameters by adding constrains of a priori information of their spatial and temporal characteristics at each epoch.

### 2.1. A priori Constraint

Usually, the ionosphere models such as the International Reference Ionosphere (IRI) model [39], Bent model [40] and Klobuchar model [41] can be used for the priori constraint. In this study, the Global Ionospheric Model (GIM) provided by the Center of Orbit Determination in Europe (CODE) is used. The ionospheric delays provided by the priori ionospheric model can be expressed as:

(1) $$ion_{i,s-prior}^{p}=M_{I_{i,s}}^{p}\times \left[-40.28/f_{s}^{2}\times VTEC_{i,s-prior}^{p}\right]$$

where, $ion_{i,s-prior}^{p}$ is the priori constrains for ionospheric delays, $M_{I_{i,s}}^{p}$ is the mapping function of the ionospheric delays. $VTEC_{i,s-prior}^{p}$ represents the total electron content in the vertical component by the prior ionospheric model, p denotes the satellite, i denotes the receiver, s is the signal channel, and f is the signal frequency.

### 2.2. Spatial Constraint

The ionosphere VTEC’s spatial behavior can be expressed as a polynomial function of the latitude differences and longitude differences in the sun-fixed reference frame [11,42]:

(2) $$ion_{i,s-space}^{p}=M_{I_{i,s}}^{p}\times \left[-\frac{40.28}{f_{s}^{2}}\times \left(\overset{n}{\underset{n_{0}=0}{\sum }}\overset{m}{\underset{m_{0}=0}{\sum }}E_{n_{0}m_{0}}{\left(\varphi -\varphi_{0}\right)}^{n_{0}}{\left(\lambda -\lambda_{0}\right)}^{m_{0}}\right)\right]$$

where, $ion_{i,s-space}^{p}$ represents the value of ionospheric space constraint, m,n are the order numbers of the polynomial, the value of the order number can be set as two. φ,λ are latitude and longitude of IPP in the sun-fixed reference frame; and $\varphi_{0},\lambda_{0}$ describe latitude and longitude of the station in the sun-fixed reference frame; $E_{n_{0}m_{0}}$ is the estimated polynomial coefficient. The coefficients can be estimated epoch wise by Equation (3) when the number of visible satellites is more than four for the BDS system. If the satellite in view is less than four, the coefficients of the last epoch are used [42].

(3) $$\overset{n}{\underset{n_{0}=0}{\sum }}\overset{m}{\underset{m_{0}=0}{\sum }}E_{n_{0}m_{0}}{\left(\varphi -\varphi_{0}\right)}^{n_{0}}{\left(\lambda -\lambda_{0}\right)}^{m_{0}})=VTEC_{i,prior}^{p}$$

### 2.3. Temporal Constraint

As the temporal variation of ionospheric VTEC is very slow, the epoch-wise variation of ionosphere delays can be modeled by a stochastic process. In this study, the temporal correlations of the DD ionospheric delay residuals are considered as a random walk process [43]. The temporal constraint of each satellite-receiver pair is described as follows:

(4) $$ion_{i,s-temp}^{p}=I_{i,s}^{p}\left(last\right)+\Delta \delta I_{i,s}^{p}$$

where, $ion_{i,s-temp}^{p}$ is the value of ionospheric temporal constraint, $\Delta \delta I_{i,s}^{p}$ represents the epoch-wise variation of the ionospheric delay.

### 2.4. The PPP Model

In our PPP model, the ionospheric delays are estimated as unknown parameters by adding constraint of a priori information, i.e., the spatial and temporal characteristics. Concerning the tropospheric delays, the Saastamoinen (SAAS) troposphere model and Global Mapping Function (GMF) are used to calculate the tropospheric error corrections, and the tropospheric residuals are estimated as random walk processes [44]. The satellite DCB is corrected by DCB files provided by the International GNSS Service (IGS), and the receiver DCB is estimated as an unknown parameter at each epoch. The satellite ephemeris errors are corrected by real-time corrections provided by the International GNSS Monitoring and Assessment Service (IGMAS) analysis center, and the receiver clock errors are estimated as white noises. The antenna parameters, Earth rotation parameters and DCB parameters are obtained from the IGS. The antenna phase center offsets (PCO) and variations (PCV) for satellites and receivers are corrected by the absolute antenna model [45]. In addition, the effects including solid tide, ocean tide, relativity effect, and phase wind up are also corrected following the descriptions in Dach et al. [44]. The PPP model can be written as follows:

(5) $$P_{i,s}^{p}=\rho^{p}+\left[trop+ion_{i,s}^{p}-DCB_{i,s}-c\delta t_{i}\right]+\left[c\delta t^{p}+DCB_{i,s}^{p}+M_{P}^{p}\right]+\varepsilon_{P,i,s}^{p}$$

(6) $$\varepsilon_{P,i,s}^{p}\sim N\left(0,\sigma_{P,i,s}^{2}\right)$$

(7) $$\Phi_{i,s}^{p}=\rho^{p}+\left[trop-ion_{i,s}^{p}-c\delta t_{i}+\lambda_{s}Amb_{s}\right]+\left[c\delta t^{p}+M_{\Phi }^{p}\right]+\varepsilon_{\Phi ,i,s}^{p}$$

(8) $$\varepsilon_{\Phi ,i,s}^{p}\sim N\left(0,\sigma_{\Phi ,i,s}^{2}\right)$$

(9) $$ion_{prior}^{p}=ion_{i,s}^{p}+\varepsilon_{prior}$$

(10) $$\varepsilon_{prior}\sim N\left(0,\sigma_{prior}^{2}\right)$$

(11) $$ion_{temp}^{p}=ion_{i,s}^{p}+\varepsilon_{temp}$$

(12) $$\varepsilon_{temp}\sim N\left(0,\sigma_{temp}^{2}\right)$$

(13) $$ion_{space}^{p}=ion_{i,s}^{p}+\varepsilon_{space}$$

(14) $$\varepsilon_{space}\sim N\left(0,\sigma_{space}^{2}\right)$$

where, P,Φ are the pseudo-range and carrier phase measurements, respectively. ρ is the geometric distance from satellite to the receiver. p represents the satellite, i represents the receiver, s denotes the frequency, λ and Amb are the wave length and phase ambiguity, respectively. M expresses the combined correction of other un-modeled errors. $ion_{prior},ion_{temp},ion_{space}$ are the virtual observations of the ionospheric priori information constraint, i.e., temporal constraint and spatial constraint. ε is the observation noise, δ is the standard deviation. Based on Equations (5)–(14), the least square is employed for data processing. When N satellites are tracked for one station at each epoch, there are 3×N+6 parameters needed to be estimated. These parameters are summarized in Table 1.

In parameters estimation, the receiver DCB, troposphere residuals and ionosphere parameters are estimated as random walk processes at each epoch. The phase ambiguity is treated as a constant in a continuous arc, which should be re-initialization when a cycle slip occurs. The measurement noise level and the satellite elevations are used to determine the observation weights. And the weights of the ionospheric virtual observations are determined by local time, noise level and satellite elevations [32,42].

## 3. The URTK Solution Model Based on Raw Observations

For the URTK with raw observations, the key issues include baseline solution of the reference stations, retrieval and interpolation of the un-differenced observation corrections, the rover’s positioning and ambiguity resolution.

### 3.1. The Baseline Solution Model for the Reference Stations with Raw Observations

For the baseline solution of the reference station, we used the DD observations by estimating the baseline components ($dX_{j},dY_{j},dZ_{j}$), DD ambiguity ($Amb_{L_{s},ij}^{pq}$) and DD atmospheric delay ($\delta I_{ij}^{pq},\delta T_{ij}^{pq}$) parameters. The linearized DD pseudo-range and carrier phase observation equations are expressed as follows:

(15) $$P_{P_{s},ij}^{pq}=\rho_{ij}^{pq}+\left(l_{j}^{q}-l_{j}^{p}\right)dX_{j}+\left(m_{j}^{q}-m_{j}^{p}\right)dY_{j}+\left(n_{j}^{q}-n_{j}^{p}\right)dZ_{j}+\frac{f_{1}^{2}}{f_{s}^{2}}\delta I_{ij}^{pq}+M_{T}\delta T_{ij}^{pq}+\varepsilon_{{P_{s}}_{ij}^{pq}}$$

(16) $$\varepsilon_{{P_{s}}_{ij}^{pq}}\sim N\left(0,\sigma_{{P_{s}}_{ij}^{pq}}^{2}\right)$$

(17) $$\Phi_{L_{s},ij}^{pq}=\rho_{ij}^{pq}+\left(l_{j}^{q}-l_{j}^{p}\right)dX_{j}+\left(m_{j}^{q}-m_{j}^{p}\right)dY_{j}+\left(n_{j}^{q}-n_{j}^{p}\right)dZ_{j}-\frac{f_{1}^{2}}{f_{s}^{2}}\delta I_{ij}^{pq}+M_{T}\delta T_{ij}^{pq}+\lambda_{s}Amb_{L_{s},ij}^{pq}+\varepsilon_{{L_{s}}_{ij}^{pq}}$$

(18) $$\varepsilon_{{L_{s}}_{ij}^{pq}}\sim N\left(0,\sigma_{{L_{s}}_{ij}^{pq}}^{2}\right)$$

where the subscripts i and j represent the base and user receivers, respectively, and the superscripts p and q represent the pair of satellites while p is the reference one. s represents the signal channel, “1” is the L1 channel. The combined subscript ij means differencing between two receivers, and the combined superscript pq denotes the differencing between two satellites. P,Φ are the pseudo-range and carrier phase observables, respectively, and λ denotes the wavelength of the carrier. The baseline components dX, dY, dZ are to be solved, and the symbols l, m and n are the unit vectors on the line-of sight from receiver to satellite. Amb is the integer ambiguity of the carrier phase, and δI and δT are the ionospheric delay and tropospheric delay, respectively. f is the carrier phase frequency, ε denotes the measurement noise, σ expresses the standard deviation of the noise, and ρ is the geometric distance from satellite to the receiver. Moreover, the mapping function $M_{T}$ for the wet components of tropospheric delay is defined as follows:

(19) $$M_{T}=\left[M_{T-i}^{p}+M_{T-i}^{q}+M_{T-j}^{q}+M_{T-j}^{q}\right]/4.0$$

In Equations (15)–(19), the least square is used for data processing. When N common satellites are tracked for one pair station at each epoch, there will be 3 × (N − 1) + 4 parameters needed to be estimated. These parameters are listed in Table 2.

For the parameters estimation, the station coordinates are tightly constrained. The troposphere and ionosphere parameters are estimated as random walk processes. The DD phase ambiguity is treated as a constant in a continuous period, which should be re-initialization when a cycle slip occurs. And the observation weights are determined by the measurement noise level and satellite elevation [32,42].

### 3.2. The Retrieval of the Un-Differenced Observation Corrections

In order to retrieve the UD observation corrections, the DD ambiguities need to be fixed firstly. The LAMBDA method is utilized here for the integer ambiguity searching [46]. The retrieval of the UD observation corrections can be divided into two steps. The first step is to recover the UD ambiguity, and the second part is to retrieve the UD observation corrections.

Based on the constraint that the DD ambiguity is integer and has been fixed, the other un-differenced ambiguities which formed the DD ambiguity can be recovered using Equation (20) when the integer part of some un-differenced ambiguities are defined as the datum in one pair of baseline:

(20) $$\left[\begin{array}{c} Amb_{i}^{p}\\ \begin{array}{c} Amb_{i}^{q}\\ Amb_{j}^{p} \end{array}\\ Amb_{j}^{q} \end{array}\right]={\left[\begin{array}{c} 1\\ \begin{array}{c} 0\\ 0 \end{array}\\ 1 \end{array}\begin{array}{c} 0\\ \begin{array}{c} 1\\ 0 \end{array}\\ -1 \end{array}\begin{array}{c} 0\\ \begin{array}{c} 0\\ 1 \end{array}\\ -1 \end{array}\begin{array}{c} 0\\ \begin{array}{c} 0\\ 0 \end{array}\\ 1 \end{array}\right]}^{-1}\left[\begin{array}{c} b_{i}^{p}\\ \begin{array}{c} b_{i}^{q}\\ b_{j}^{p} \end{array}\\ Amb_{ij}^{pq} \end{array}\right]$$

Here, $b_{i}^{p},b_{i}^{q},b_{j}^{p}$ are the selected UD integer ambiguity datum. $Amb_{i}^{p},Amb_{i}^{q},Amb_{j}^{p},Amb_{j}^{q}$ are the recovered UD integer ambiguities. $Amb_{ij}^{pq}$ is the fixed DD integer ambiguity.

By transferring the datum ambiguities of the reference satellites through different satellite pairs, the other un-differenced ambiguities of this baseline can be recovered. Then, by the transfer of datum ambiguities of the reference stations through different baselines, all the un-differenced ambiguities of the whole network can be recovered. It is noteworthy that the selected UD ambiguities datum must be consistent for the same network.

After all UD ambiguities are recovered, the UD observation corrections can be calculated using the following equations:

(21) $$omcP_{i,s}^{p}=P_{i,s}^{p}-\rho_{i,s}^{p}-c\delta t_{i,s}+c\delta t^{p}+Model_{P_{i,s}}^{p}$$

(22) $$omc\Phi_{i,s}^{p}=\lambda \Phi_{i,s}^{p}-\rho_{i,s}^{p}+\lambda Amb_{i,s}^{p}-c\delta t_{i,s}+c\delta t^{p}+Model_{\Phi_{i,s}}^{p}$$

Here, Model describes the modeled errors, such as the tropospheric errors, relativity errors, phase center offset error and phase wind up. omc is the comprehensive correction, it contains the orbit residual, clock residual, Uncalibrated Phase Delay (UPD), DCB and some other un-modeled errors.

### 3.3. The Interpolation of the UD Observation Corrections

At the user end, three nearby reference stations are selected as the augmentation stations. Based on these UD observation corrections from the augmentation, we can calculate the rover’s corrections using Equation (23):

(23) $$omc_{s,u}^{p}=a\left(omc_{s,2}^{p}-omc_{s,1}^{p}\right)+b\left(omc_{s,3}^{p}-omc_{s,1}^{p}\right)+omc_{s,1}^{p}$$

(24) $$a=\frac{(x_{3-}x_{1})\left(y_{u}-y_{1}\right)-(x_{u}-x_{1})\left(y_{3}-y_{1}\right)}{\left(x_{3}-x_{1})\left(y_{2}-y_{1}\right)-(x_{2}-x_{1})(y_{3}-y_{1}\right)}$$

(25) $$b=\frac{(x_{u}-x_{1})\left(y_{2}-y_{1}\right)-(x_{2}-x_{1})\left(y_{u}-y_{1}\right)}{\left(x_{3}-x_{1})\left(y_{2}-y_{1}\right)-(x_{2}-x_{1})(y_{3}-y_{1}\right)}$$

Here, 1,2,3 represents the augmentation stations, u refers to the rover station, x,y are the plane coordinates of each station.

### 3.4. The URTK Model

At the user end, the observation corrections can be interpolated using Equation (23) at first. Then the UD observations are corrected and the positioning is performed. The positioning model at the user end can be written as:

(26) $$P_{i,s}^{p}=\rho_{i}^{p}+l_{i}^{p}X_{i}+m_{i}^{p}Y_{i}+n_{i}^{p}Z_{i}+\left[DCB_{F}-c\delta t_{i}\right]+\left[omcP_{i,s}^{p}\right]+Model_{P_{i,s}}^{p}+\varepsilon_{P_{i,s}^{p}}$$

(27) $$\varepsilon_{P_{i,s}^{p}}\sim N\left(0,\sigma_{P_{s}}^{2}\right)$$

(28) $$\Phi_{i,s}^{p}=\rho_{i}^{p}+l_{i}^{p}X_{i}+m_{i}^{p}Y_{i}+n_{i}^{p}Z_{i}+\left[\lambda_{s}Amb_{s}^{p}-c\delta t_{i}\right]+\left[omc\Phi_{i,s}^{p}\right]+Model_{\Phi_{i,s}i}^{p}+\varepsilon_{\Phi_{i,s}^{p}}$$

(29) $$\varepsilon_{\Phi_{i,s}^{p}}\sim N\left(0,\sigma_{\Phi_{s}}^{2}\right)$$

Using Equations (26) and (29), the least square is employed for the estimation. The estimated unknown parameters are the positioning components ($X_{i},Y_{i},Z_{i}$), ambiguity ($Amb_{s,i}^{p}$) and the combined receiver clock error $\delta t_{i}$. The clock error is estimated at each epoch as a white noise. The observation corrections include the DCB and UPD of both satellites and receivers at the reference stations. For the user positioning, the satellite UPD can be cancelled when using the observation corrections, but the receiver DCB still need to be estimated. Meanwhile, the receiver UPD cannot be eliminated, thus the ambiguity is not integer. Fortunately, the Single-Differenced (SD) projection can be applied for the ambiguity resolution to realize the fast convergence and high-precision positioning.

## 4. The Integration of PPP and URTK Based on Raw Observations

### 4.1. An Integrated Model

In order to integrate PPP and URTK based on raw observations into a seamless positioning service, the key point is to develop a unified model of PPP and URTK. The unified model of PPP and URTK can be written as follows:

(30) $$P_{i,s}^{p}=\rho^{p}+\left[Estimation\right]+\left[Correction\right]+\varepsilon_{P,i,s}^{p}$$

(31) $$\varepsilon_{P,i,s}^{p}\sim N\left(0,\sigma_{P,i,s}^{2}\right)$$

(32) $$\Phi_{i,s}^{p}=\rho^{p}+\left[Estimation\right]+\left[Correction\right]+\varepsilon_{\Phi ,i,s}^{p}$$

(33) $$\varepsilon_{\Phi ,i,s}^{p}\sim N\left(0,\sigma_{\Phi ,i,s}^{2}\right)$$

(34) $$Virtual=\left[Estimation\right]+\varepsilon_{v,i,s}^{p}$$

(35) $$\varepsilon_{v,i,s}^{p}\sim N\left(0,\sigma_{v,i,s}^{2}\right)$$

Here, “Estimation” consists of the unknown parameters such as the coordinates of the receiver, phase ambiguity Amb, troposphere delay trop, ionosphere delay $ion_{s}^{p}$, hardware delay $DCB_{s}$ and the receiver clock error $\delta t_{i}$. “Correction” represents the corresponding correction parts in Equations (5)–(8), (21) and (22). Moreover, in terms of the integrated model for the URTK solution, the ionosphere estimates are the residuals. Thus, the values of ionosphere virtual observations can be set to zero for a regional area, i.e., Virtual=0.

### 4.2. Data Processing Flow

**Figure 1:**
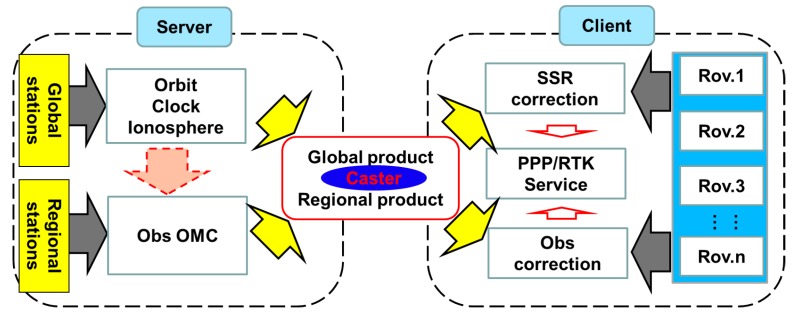
The processing flow of integrated PPP/RTK algorithm (“Rov.” represents the rover stations, “Obs” represents the observation domain, “SSR” denotes the state space represents, “OMC” is the observed minus the computed corrections, “Caster” is used to receive and broadcast the global and the regional products).

The data processing is shown in Figure 1, which consists of two parts: the server and the user end. At the server end, the global reference stations are processed in real-time to generate the precise orbit, clock and ionosphere products, while the regional reference stations are processed in real-time to generate the OMC corrections. Both the global and regional products are broadcasted to the users by the caster.

At the user end, when they are global users, the satellite orbit and clock errors are corrected by the received corrections, and the other modeling errors are corrected by using the empirical models. Moreover, the un-differenced and un-combined solution models for the absolute positioning are used to get the PPP solutions. For the regional users, all the observation errors are considered as the comprehensive errors, which are corrected in the observation domain. Besides, the un-differenced and un-combined solution models for the absolute positioning are applied to achieve the URTK solutions.

## 5. Validations and Results

In order to assess the proposed algorithm for BDS wide area and local area augmentation positioning, a series of experiments were carried out in Xi’an. The data processing was carried out using the GNSS software package developed by our research group, which is capable of providing both network solution and PPP/RTK services [32]. Although its old version is only applicable to the GPS measurements, this software has been recently developed and is now able to handle with the BDS data. In the following, the results based on these experiments will be presented.

### 5.1. Data Description

**Figure 2:**
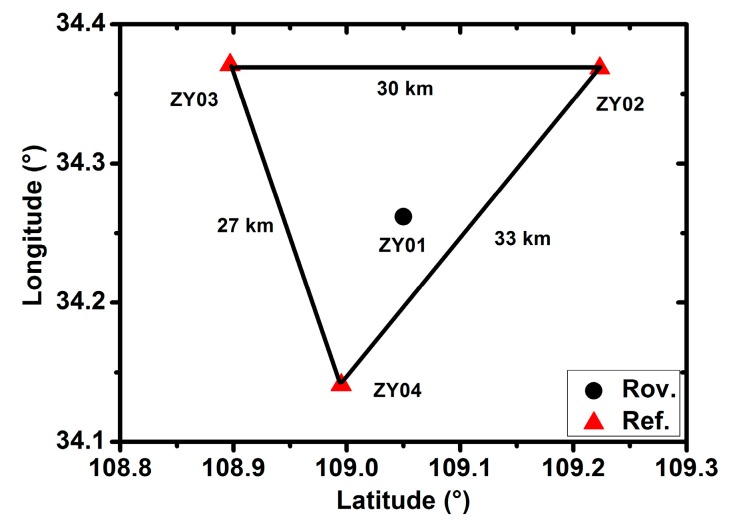
The distribution of the collected reference and rover stations (The reference stations are shown in red, and the rover station is shown in black).

The BDS observations are collected on day of year (DOY) 080 to 083 in 2016.The antenna type is HG-GOYH7151 and the receiver type is UR380, which is capable of tracking the BDS B1, B2 and B3 signals. The sampling rate is 1 s, observing from UTC 0:0:00 to 23:59:59. The data are processed in a simulated real-time mode, which indicates that all the raw observations were input to the software package epoch by epoch similar to the real-time stream, and the station positions are estimated and output epoch by epoch. The observation signals of B1 and B2 are used for the dual-frequency solution, while those of the B1 are used for the single-frequency solution. The broadcast ephemeris products are utilized here. The stations are located in the city of Xi’an in Shan Xi Province. Figure 2 shows the distribution of the collected stations, where three stations marked in red are selected as the reference stations (i.e., NT02, NT03, NT04) and the station marked in black is selected as the rover station (i.e., NT01). The distances for the three baseline pairs (NT04-NT03, NT04-NT02 and NT03-NT02) are 30, 33 and 27 km, respectively. The precise satellite orbit, clock, ionosphere and DCB products are provided by the IGMAS and used in a simulated real-time mode. The antenna PCO and PCV parameters, as well as the Earth Rotation Parameters (ERP) files are provided by the IGS.

### 5.2. Accuracy Validation

The collocated data were processed using the unified model with four different strategies in simulated real-time modes: (a) Using the dual-frequency (B1 and B2) observations and real-time orbit, clock products, processed in a static mode (It is equal to static PPP solution); (b) Using the dual-frequency (B1 and B2) observations and real-time orbit, clock products, processed in a dynamic mode (It is equal to dynamic PPP solution); (c) Using the dual-frequency (B1 and B2) observations and real-time UD OMC corrections, processed in a static mode (It is equal to static URTK solution); (d) Using the dual-frequency (B1 and B2) observations and real-time UD OMC corrections, processed in a dynamic mode (It is equal to dynamic URTK solution). The reference coordinates of these stations are calculated by using the static GNSS-PPP of three-day observations, the coordinate accuracy is better than 5 mm in horizontal and 1 cm in vertical [42].

**Figure 3:**
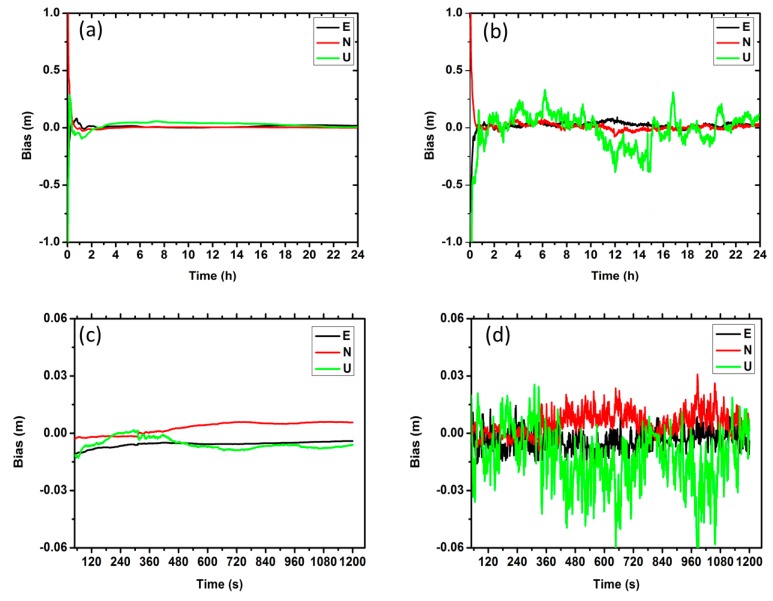
The positioning errors of the BDS PPP/URTK ((**a**,**b**) represents the static and dynamic results by PPP mode; and (**c**,**d**) represents the static and dynamic results by URTK mode).

Figure 3 shows the comparison of positioning errors for different solution strategies. We can notice that the results of dynamic model show larger noise than the static model, especially in the vertical component. In Table 3, the statistics of the positioning accuracy for four-day solutions is presented. For the PPP, the Root Mean Square (RMS) is (8, 6, 15) mm and (25, 16, 56) mm in East, North and Up components for the static and dynamic solutions, respectively. For the URTK, the RMS is (6, 5, 14) mm and (21, 17, 30) mm in East, North and Up components for the static and dynamic solutions, respectively.

### 5.3. Convergence Time

In order to make a test on the convergence time, the estimation of ambiguity parameter is reinitialized every half an hour for the PPP solution and every five seconds for the URTK solution. The criterion for the convergence is defined as that the bias of each horizontal component is smaller than 10 cm for PPP and smaller than 3 cm for URTK.

**Figure 4:**
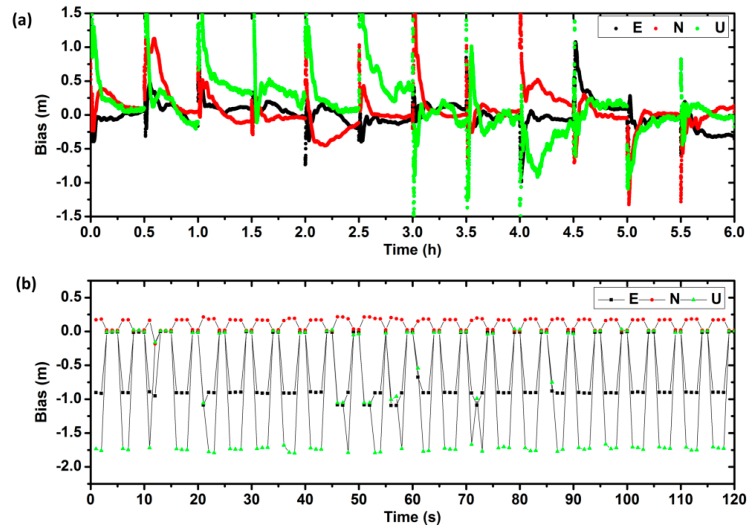
The convergence of the PPP/RTK ((**a**,**b**) represents the results of PPP and URTK, respectively).

Figure 4 shows the solution convergence of the test. It can be seen that the convergence speed of URTK model is much faster than that of the PPP model. It may be due to the fact that the common errors are precisely corrected by the observation corrections and the satellite to satellite single-differenced ambiguity can be easily fixed by URTK. In addition, Table 4 summarizes the average convergence time for the four days. The mean convergence time is about 1500 and 1.7 s for the PPP and URTK model, respectively.

### 5.4. Observation Residuals

**Figure 5:**
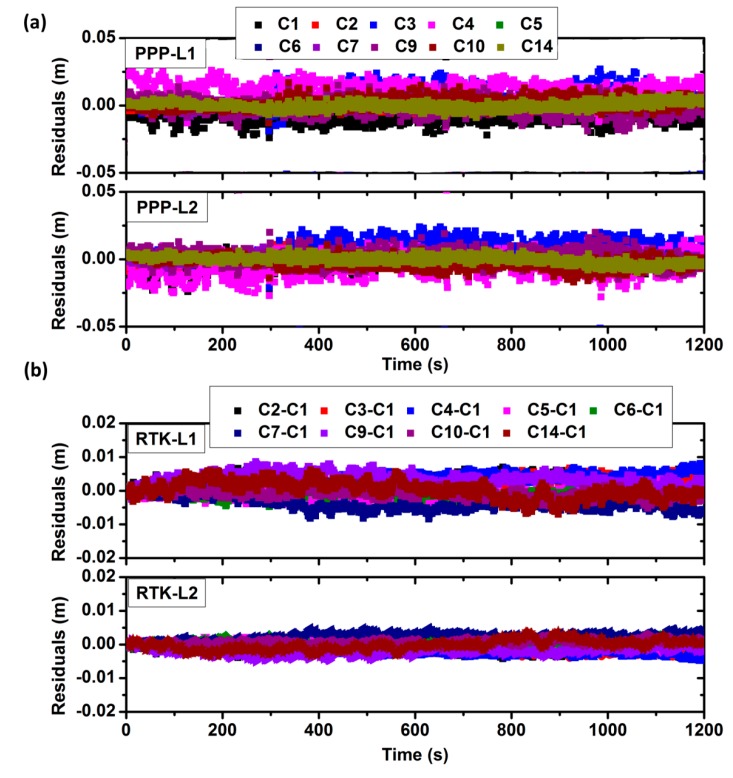
The comparison of observation residuals. (**a**) The residuals of the L1 and L2-frequency carrier phase observations by PPP mode; (**b**) The residuals of the L1 and L2-frequency carrier phase observations by URTK mode.

Figure 5 shows the comparison of observation residuals. For the URTK model, the residuals are at the level of several mm. But for the PPP model, the residuals are a little dispersive. The Standard Deviation (STD) is about 1–2 cm, which means that the errors correction in the URTK model is much better than in the PPP model. Thus the position errors for the PPP model are also much larger than for the URTK mode.

### 5.5. Ionospheric Delay

**Figure 6:**
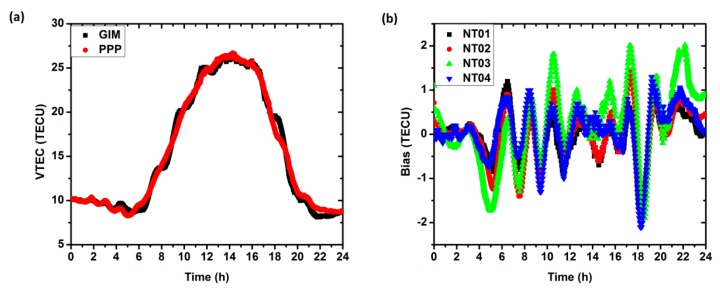
(**a**) The time series of VTEC at station “NT01” on day of 080 for the grid ionosphere model (black) and the PPP solution (red); (**b**) The differences of VTEC between the grid ionosphere model and the PPP solution for the four selected stations.

In this study, the ionospheric delay is treated as an unknown parameter and estimated together with other positioning parameters. It is also of benefit for retrieving the ionospheric VTEC. For each station, the estimated ionospheric delay can be used to establish a polynomial ionospheric model like Equation (2), thus the VTEC at the station can be obtained. As shown in Figure 6a, the VTEC gained by the PPP solution agree well with the Grid Ionosphere Model (GIM), showing the same variation trend. Figure 6b shows the differences of the VTEC between the grid ionosphere model and the PPP solution. The largest difference of the two solutions is smaller than 2 TECU. Compared to the traditional approach which usually uses the pseudo-range or carrier phase smoothed pseudo-range, our proposed method directly makes use of the carrier phase observations based on the easily operated PPP technology. It provides an alternative way for the ionosphere monitoring and modeling.

### 5.6. Analysis of Receiver DCB

For precise positioning based on raw observations, both satellite and receiver DCB must be considered. In this study, the satellite DCB are corrected using the published DCB files, and the receiver DCB are estimated as unknown parameters at each epoch.

**Figure 7:**
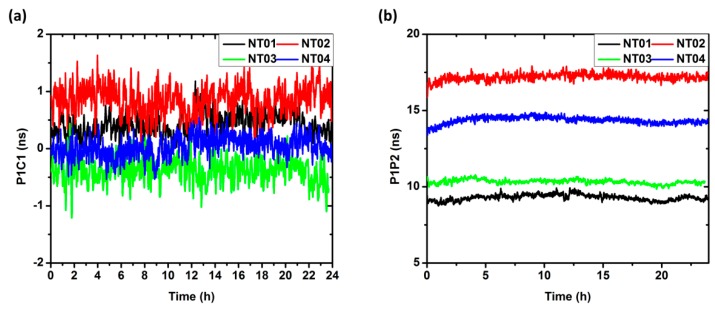
The DCB time series of (**a**) P1C1 and (**b**) P1P2 for the four selected stations.

Figure 7 shows time series of the estimated receiver DCB. It can be seen that the daily variation of the receiver DCB is very small, with a mean STD of 0.15 ns and 0.18 ns for P1C1 and P1P2, respectively. The DCB corrections for the P1C1 are very small, but are much larger for the P1P2. As its magnitude can reach several nanosecond or even larger, the DCB correction plays a significant role for the single-frequency PPP initialization that utilizes the raw observations, and it also critical for the ionosphere estimation.

## 6. Discussion and Conclusions

In this contribution, we have proposed a unified model for BDS wide area and local area augmentation positioning based on raw observations. In this model, the users can realize both PPP and RTK service. When the users are within the local reference network, the regional observation corrections can be utilized in order to achieve the fast and high precision RTK service, with a convergence time of few seconds and a precision of about 2–3 cm. When the users are out of the local reference network, the global broadcast SSR corrections can be applied to realize the PPP service, achieving a convergence time of 25 min and a positioning accuracy of 10 cm.

The proposed method was realized and validated with the BDS data collocated at four regional stations from DOY 080 to 083 in 2016. In this unified model, the PPP and NRTK can be integrated into a seamless positioning service, which is able to provide an accuracy of about 10 cm anywhere and upgrade to a few centimeters within several seconds with the support of a regional network. Meanwhile, the ionosphere VTEC and DCB values that are important for the ionosphere monitoring and modeling can be recovered.

Up to now, the multi-system and multi-frequency satellite navigation systems are under rapid development. In future, we may focus on studying the combined PPP/RTK algorithm utilizing BDS, GPS, GLONASS and Galileo systems, so as to improve the accuracy, continuity and reliability of the precise positioning. Meanwhile, applying the estimated ionosphere information to establish a high-precision and high-resolution ionosphere model is also one of the focuses for our future work. The influences of the solar cycle, seasonal and local-time dependent ionospheric variability are also needed to be studied later.

Furthermore, this study is focusing on real-time positioning, not only for PPP but also for URTK. Some previous studies have proposed the “Virtual RINEX”, which is basically NRTK for post-processing applications [47,48]. Virtual RINEX data is of potential benefits to both everyday and upmarket boutique users. For the everyday users, it can provide a vital backup that enables workable results in those annoying communications black spot areas. Besides, it also provides the potential of forward and reverses post-processing to minimize the effects of GNSS data gaps and maximize the performance. Thus, the precision, availability and reliability achieved with the Virtual RINEX will be much better than the rea-time users. For the further study, it is also meaningful to use our approach to generate the Virtual RINEX for the post-processing applications.

## Figures and Tables

**Table 1:** The classify of the estimated parameters for PPP based on raw observations.

System	Group 1	Group 2	Group 3	No.
BDS	X,Y,Z,T,	$Amb_{L_{1}}\left(N\right)$	I(N)	3 × *N* + 6
	DCB,clk	$Amb_{L_{2}}\left(N\right)$		

**Table 2:** The classify of the estimated parameters for DD baseline solution.

System	Group 1	Group 2	Group 3	No.
BDS	dX,dY,dZ	$Amb_{L_{1}}\left(N-1\right)$	δI(N−1)	3×(N−1)+4
	δT	$Amb_{L_{2}}\left(N-1\right)$		

**Table 3:** Statistics of the positioning accuracy.

Strategy	Components	Static (m)	Dynamic (m) RMS
PPP	North	0.008	0.025
	East	0.006	0.016
	Up	0.015	0.056
URTK	North	0.006	0.021
	East	0.005	0.017
	Up	0.014	0.030

**Table 4:** The statistics of the average convergence time (Unit: s).

DOY	080	081	082	083 Average
PPP	1460	1520	1540	1485
RTK	1.6	1.7	1.8	1.7
